# Open-source workflow design and management software to interrogate duckweed growth conditions and stress responses

**DOI:** 10.1186/s13007-023-01065-3

**Published:** 2023-09-01

**Authors:** Madeline Scott, Orlando de Lange, Xavaar Quaranto, Ryan Cardiff, Eric Klavins

**Affiliations:** Department of Electrical and Computer Engineering, University of Washington, Seattle, USA

## Abstract

Duckweeds, a family of floating aquatic plants, are ideal model plants for laboratory experiments because they are small, easy to cultivate, and reproduce quickly. Duckweed cultivation, for the purposes of scientific research, requires that lineages are maintained as continuous populations of asexually propagating fronds, so research teams need to develop optimized cultivation conditions and coordinate maintenance tasks for duckweed stocks. Additionally, computational image analysis is proving to be a powerful duckweed research tool, but researchers lack software tools to assist with data collection and storage in a way that can feed into scripted data analysis. We set out to support these processes using a laboratory management software called Aquarium, an open-source application developed to manage laboratory inventory and plan experiments. We developed a suite of duckweed cultivation and experimentation operation types in Aquarium, which we then integrated with novel data analysis scripts. We then demonstrated the efficacy of our system with a series of image-based growth assays, and explored how our framework could be used to develop optimized cultivation protocols. We discuss the unexpected advantages and the limitations of this approach, suggesting areas for future software tool development. In its current state, our approach helps to bridge the gap between laboratory implementation and data analytical software for duckweed biologists and builds a foundation for future development of end-to-end computational tools in plant science.

**Supplementary Information:**

The online version contains supplementary material available at 10.1186/s13007-023-01065-3.

## Background

Scientific interest in the diminutive freshwater plants of the Lemnaceae family has grown in recent years [1]. Duckweed have demonstrated applications in phytoremediation of heavy metals [2–4], and also provide valuable protein for fish in aquaculture [5]. Laboratory research into duckweed is motivated in part by investigating industrial and agricultural applications; however, duckweeds are also re-emerging model species for laboratory plant science since they are small, easy to cultivate, and reproduce quickly [6, 7]. In this study we present novel scripts to integrate with existing laboratory software platforms and provide examples of how these scripts can be used to support laboratory research work with duckweeds.

Controlled laboratory growth assays have proven to be powerful tools to investigate duckweed physiology and evolutionary biology [8, 9], as well as a means to detect and remediate toxins in freshwater [10]. Metrics such as relative growth rate, doubling time, and relative weekly yields can be calculated by comparing fresh weights of duckweed at the start and end of the experiment [11]. It has become increasingly common to collect and analyze time-series of top-down images of duckweed growing in liquid as an alternative or additional method of measuring growth rates [12–15]. Duckweed float and tend to spread out in two dimensions across the water surface as they expand and produce new daughter buds, and as such the rate of surface area coverage by a duckweed population correlates linearly with the rate of mass accumulation [16].

Since duckweeds rarely flower or set seed [17], each laboratory duckweed genotype is generally maintained as an asexually propagating lineage. Growth rates of duckweed vary between genotypes and environmental conditions [18–20]. To our knowledge, no characterizations of the relationship between cold storage duration and subsequent viability and growth rates exist. Therefore, laboratories working with multiple duckweed varieties must maintain each line through regular transfer to fresh media according to a unique schedule. Contamination is a constant risk if duckweed lineages are being maintained in axenic culture for laboratory experiments [21]. Personnel in a duckweed research laboratory need to manage the organizational work of maintaining duckweed lines as well as minimizing and troubleshooting contaminations, and establishing protocols to suit local conditions and genotypes of interest.

Plant scientists have increasingly been designing, sharing, and making use of sophisticated, often automated, analysis tools to process large datasets composed of images of plants and using these to identify, characterize and compare phenotypes of interest [22]. Compared to start and end point weight measurements, image time-series provide resolution of growth dynamics within the time course as well as the possibility of gaining insight into physiological and development factors inferred from frond size, shape and color [22]. A wide array of open-source plant image analysis packages have been developed to support image-based plant biology research workflows [22], including tools developed specifically for duckweed [13, 23, 14]. To make use of these tools, researchers need to be able to keep large numbers of image files organized along with relevant metadata for analysis, introducing a logistical challenge.

We set out to develop software tools to support duckweed research workflows that involve maintenance of genotypes and quantification of growth rates using time-series images. To do this, we built off of the existing Aquarium open-source laboratory management system previously developed in our lab to support synthetic biology workflows [24]. Aquarium incorporates LIMS functionalities as well as a GUI-based workflow design and support for protocol execution with instructions provided as just-in-time graphics at the lab bench. We created novel protocol scripts for Aquarium using the domain-specific language Krill, as well as compatible Python-based data analysis scripts relying on existing packages, particularly OpenCV2 [25].

The Aquarium protocol scripts and our novel data analysis scripts combined provide solutions to two specific problems for duckweed laboratory researchers, the first of which is managing the maintenance of multiple separate lineages of asexually reproducing organisms for which long-term cold storage protocols are not available. Researchers can use Aquarium to keep track of the history and cultivation requirements of their duckweed stocks to ensure experimental consistency. Our open-source software also addresses the problem of managing datasets, including metadata, for experiments that involve the collection of large numbers of image files. Our scripts demonstrate how Python can interface with Aquarium to easily analyze large data sets of images.

Digital infrastructure for image processing requires physical infrastructure for sample handling and image collection, and there has been a corresponding interest in developing and disseminating automated imaging systems [26–28]. Several systems have been published to support scripted analysis of growth rates of duckweeds *Lemna minor* [12, 13] and *Spirodela polyrhiza* [14]. While there has been significant research interest paid to the development of novel hardware and software systems, there has not been a corresponding attention paid to the laboratory management infrastructure needed to support researchers operating high throughput or automated imaging workflows. We set out to explore whether Aquarium could be a suitable system to address challenges related to the use of scripted image analysis workflows in plant science. In particular we were interested in workflow planning and task management as well streamlining data management and the pipeline from collection to scripted analysis.

We addressed both of these challenges—managing duckweed stocks and large datasets—by developing and testing a suite of Aquarium types, the core database objects of Aquarium, including inventory types and workflow elements (operation types—OTs). Inventory types include sample types, which are unique categories of biological entity (e.g. a duckweed genotype) and containers, which are types of items that can be manifestations of a biological entity (e.g. dish of duckweed). OTs contain many elements, including a definition of the input and output sample types and containers as well as a protocol script written in Krill, a domain specific language derived from Ruby on Rails. We developed a suite of Aquarium OTs to provide a modular framework from which a range of different duckweed cultivation and research workflows can be constructed. Since the Aquarium system has been previously described, we focus here on the novel software written to support duckweed work, the guidelines we have developed for implementation, and examples from real world tests conducted in our lab.

The OTs and associated protocol code were developed and updated iteratively over eighteen months, while being used to facilitate continuous maintenance of duckweed cultures and a range of different growth assays, by a collaborative team of one postdoctoral researcher and one or two undergraduate or graduate students at any given time. Based on this experience we refined the set of OTs and carried out the test case experiments described below. Details of those experiments are shared below. Experimental data and analysis scripts can be downloaded from the Github directory (https://github.com/mtscott321/duckweed_data_analysis). The novel aquarium code as well can be downloaded from the supplementary material (Additional file 1) along with a README guide (Additional file 2) and a PDF with vignettes illustrating the user interface for working with Aquarium to adapt and deploy the workflow elements we have developed in this study (Additional file 3).

## Results

### Supporting cultivation and stock maintenance

**Fig. 1:**
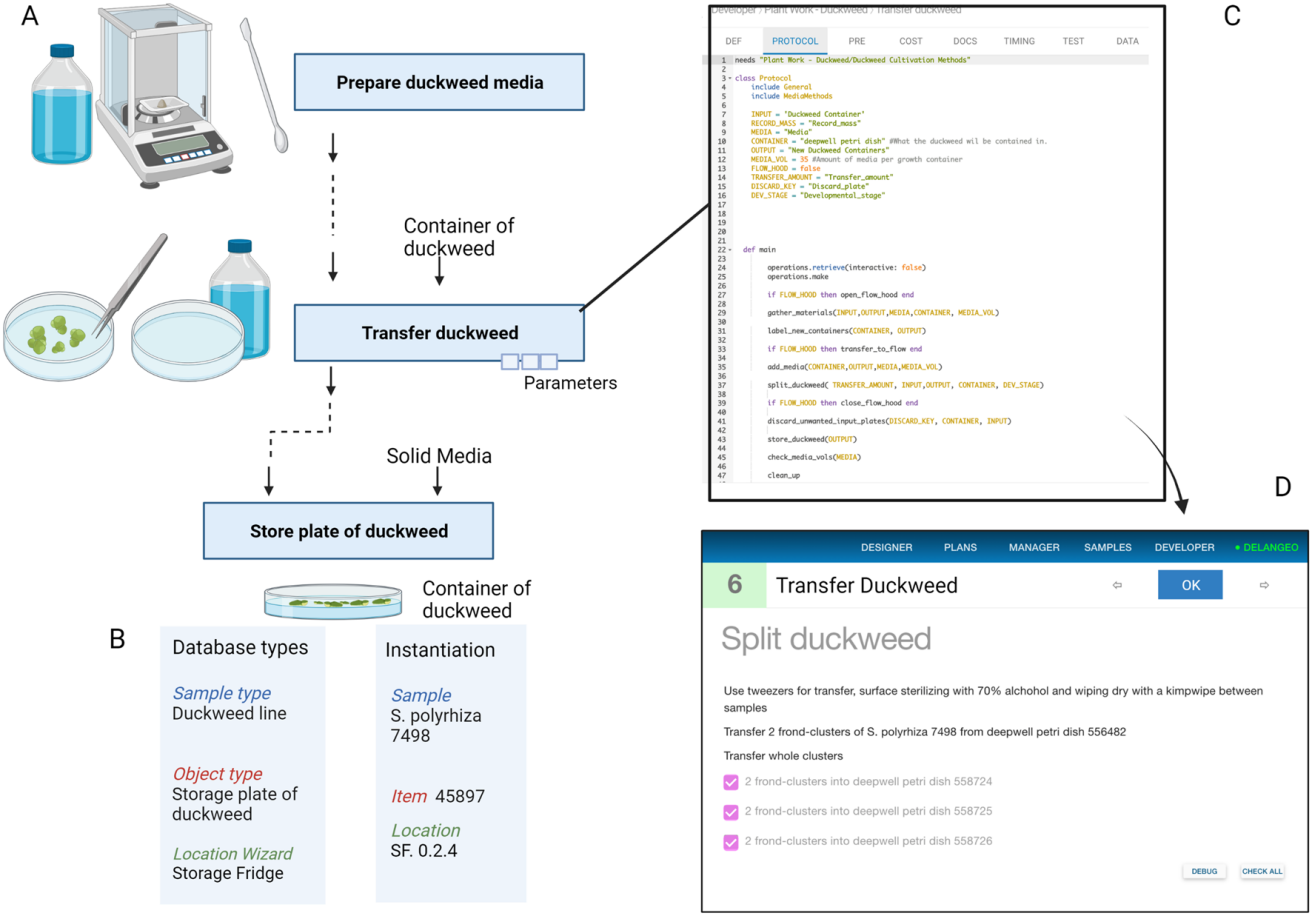
Graphical summary of the core operation types for duckweed stock maintenance. Laboratory protocols (**A**) can be represented in Aquarium as sets of defined Types that can be instantiated as real-world laboratory inventory and locations (**B**). Each OT (e.g. “Transfer duckweed”) in panel A has a corresponding protocol code script (**C**) written in the Krill programming language using the Integrated Development Environment within the Aquarium web application. When a plan (connected series of operations) is created and launched, the individual operations can be run as Jobs with step-by-step onscreen instructions (**D**) provided to technicians at the lab bench according to the specifications within the protocol code

One of the drawbacks of duckweeds as laboratory organisms is that most will not flower under standard laboratory cultivation conditions. Additionally, seed harvesting is a demanding process due to the diminutive size of their fruits [17]. Therefore, duckweed stocks must be actively maintained and regularly transferred to fresh media, akin to splitting cell cultures. We developed a set of Aquarium OTs for duckweed stock maintenance and management as well as a database context for these modules consisting of sample Types, containers (object types), and location wizards [24]. The set up is outlined in Fig. 1A, and centers around maintaining duckweed in discrete culture vessels, which we implemented locally as deepwell petri dishes but could be flasks or other containers. A key feature of our approach is to define each duckweed genotype as a Sample with items such as *Container of Duckweed* belonging to the duckweed genotype sample of the plants within them (Fig. 1B).

Operation types (OTs) consist of a name, text description, specific inputs and outputs, and a protocol script written in Krill. An example of Krill code and how this renders for laboratory technicians are shown in Fig. 1C and D. Optional additions include a separate script to define preconditions and a cost model. OTs are created within the Aquarium Integrated Development Environment (IDE) and then (once made live) they are available to be integrated into plans. Each instance of an OT within a plan is an operation and specific inventory database objects must be specified for input and output fields before the plan can be launched. The rectangles with text in Fig. 1A mirror the graphic display in the Aquarium designer interface, with graphics added to illustrate the key items and processes involved in executing the OTs. Illustrative screenshots can be found in Additional file 3. In total, we created eleven novel OTs for the work in this paper. These can be downloaded as a bundled “.aq” file from the supplementary material accompanying this manuscript (Additional file 1). To view and interact with the code we suggest installing a local Aquarium instance, following the instructions provided at http://www.aquarium.bio/ or in Additional file 2.

**Fig. 2:**
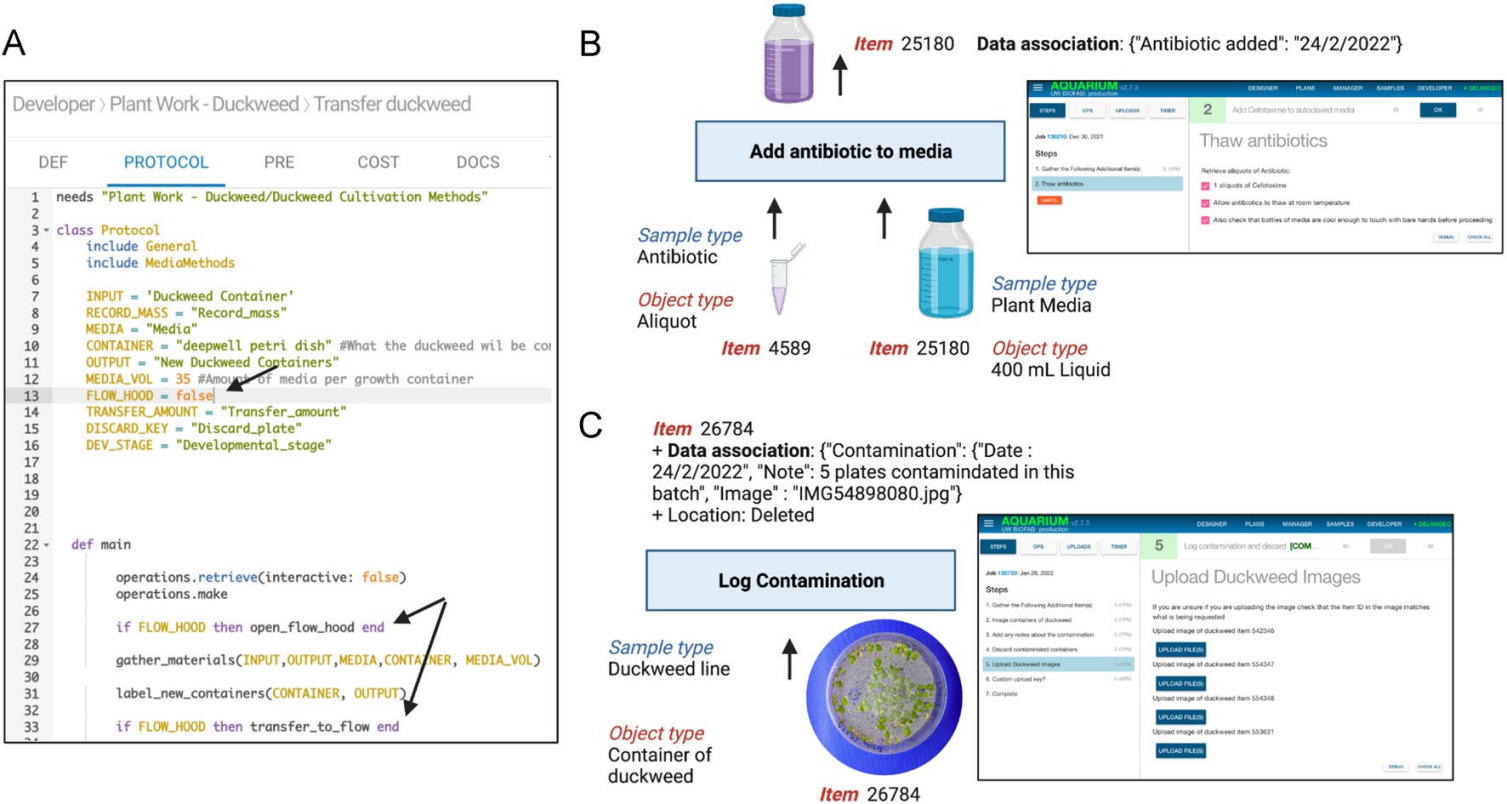
Minimizing and troubleshooting contamination. **A** Within the “Transfer duckweed” operation type a constant (“FLOW_HOOD” indicated by arrow) can be toggled between true or false depending on the needs of a particular laboratory. The value of this constant is used to hide or display specific instruction sets when the protocol is run (e.g. code lines indicated by bifurcated arrow). An optional operation type (**B** “Add antibiotic to media”) generates instructions to add an appropriate amount of a defined antibiotic into the media and to add a metadata tag to the media item that antibiotic has been added. As shown in panel (**C**), duckweed containers found to be contaminated can be entered as inputs to an operation (“Log Contamination”, blue rectangle) that, when run, will prompt the technician to upload an image (screenshot of technician interface to the right) as well as additional notes and then discard the plate. This operation produces no output item (arrow above operation) but does lead to data associations being added to the relevant item in the Aquarium database, including an entry that can be used to pull the relevant image file using a Python data analysis script

Contamination is a significant concern for those maintaining laboratory duckweed stocks. Stocks are generally maintained in axenic culture [11]. Sugar is often added to media to promote more rapid growth of duckweed, but at the same time this increases the risk of microbial colonization. To provide options to reduce the risk of contamination we added code to instruct technicians to work within a lateral flow hood for all of our duckweed handling OTs that can be easily turned on or off during local implementation (Fig. 2A). We also implemented standard precautions within our workflow modules, including an OT to facilitate addition of an antibiotic to liquid media (Fig. 2B). Finally, wecreated an OT that can be run when contamination is encountered to log the details and upload an image before discarding the contaminated item in order to facilitate future troubleshooting efforts and to be able to track contamination rates over time (Fig. 2C).

**Fig. 3:**
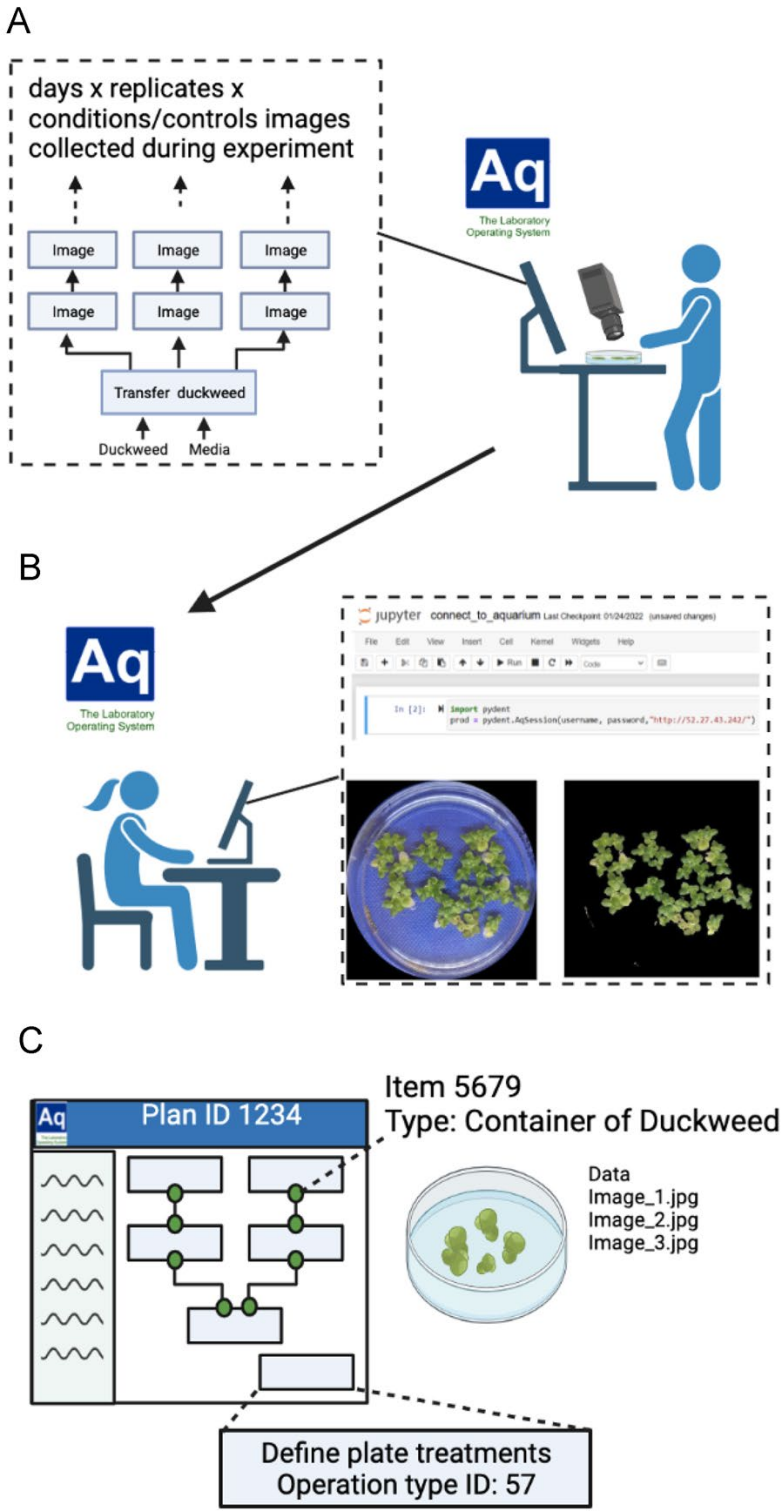
Overview of growth assay workflow including laboratory execution and data analysis. A technician at the lab bench is guided through the steps of collecting images of containers of duckweed according to the plan launched for the experiment (**A**). The images are uploaded via an on-screen prompt by the technician as part of the Aquarium job. The technician takes an image of the plate and uploads it to Aquarium. A Jupyter notebook guides the user through the process of identifying the plate borders, duckweed in the plate, and quantifying percentage coverage (**B**). In the strongly typed Aquarium LIMS, all objects have a unique ID as well as a number of possible properties with unique names (**C**). This system is the basis for the design of analysis scripts using the Trident API. For example, our data analysis script relies on user input of a specific plan ID to identify an experiment, then image data are extracted by performing a scripted search for Items of type ‘Container of Duckweed’ and pulling all data associations from each item. We also include an operation of type ‘Define plate treatment’ in each experiment plan to store important metadata, extracted in a similar way using the unique name or ID of the operation type to find it within the operations associated with the plan of interest

Duckweed growth assays typically measure the increase in mass or frond area over time under defined conditions [11]. Increasingly, researchers are making use of computational image analysis to calculate growth rates from time course image sets [12–14]. Aquarium can support time-series image collection workflows by guiding technicians to collect data, providing a database structure for data management and the use of the Trident API to provide scripted retrieval of data and metadata from an Aquarium instance to feed into data analysis. We therefore created OTs for collecting image data as well as for the collection of fresh and dry weight (Fig. 3A), and developed a set of Python scripts for data analysis, drawing in particular on tools within the pydent, opencv and numpy packages (Fig. 3B). Individual images are tagged with a date and with the unique ID of the duckweed item they represent (Fig. 3C) allowing for rapid, scripted analysis of folders of collected images. Summaries of the protocols within each of the data collection operation types are provided in the Methods above, and all Aquarium code and Python scripts can be found in the Github repository www.github.com/mtscott321/duckweed_data_analysis.

**Fig. 4:**
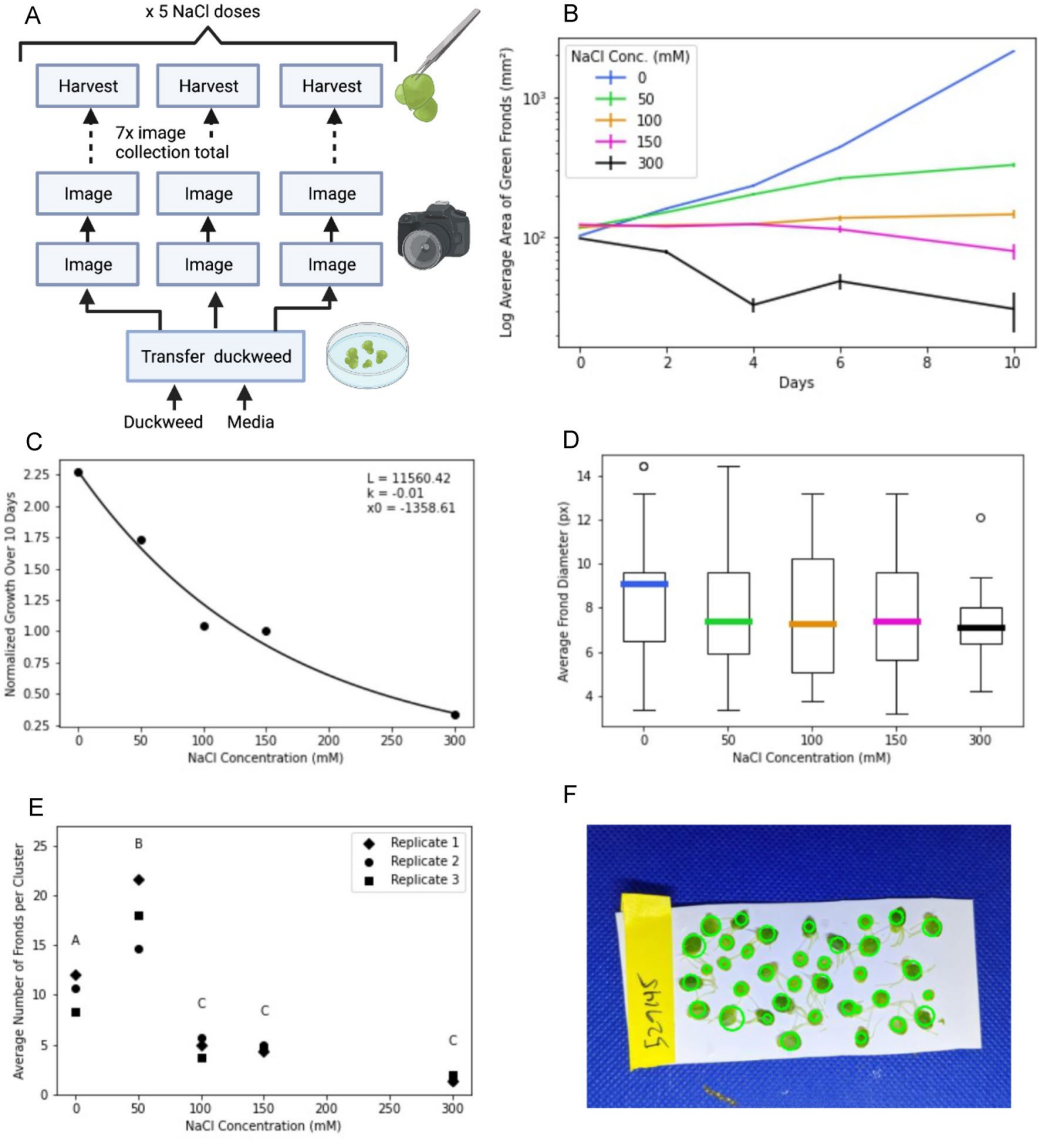
Growth responses of duckweed to varying doses of salt. An overview of experimental design is shown in (**A**), mirroring the box (operations) and line (items) scheme used in the Aquarium designer interface. Duckweed fronds were grown in deepwell petri dishes for 10 days in media supplemented with 0–300 mM sodium chloride over 10 days. Images were collected approximately every 2 days and analyzed to quantify the area of green fronds in the dish. The resulting growth curves are shown plotted on a log scale in panel (**B**). After the 10-day trial, the growth was normalized by dividing the final area by the initial area for each sodium chloride concentration. A Hill equation was fit to the data to create a dose–response curve as shown in (**C**). After 10 days, fronds were harvested and the individual fronds from a sample of 5 ramets from each dish were manually separated from one another. An image was taken of the manually separated fronds and used to calculate frond diameters for plants in each of the treatment groups (**D**), as well as average number of fronds per ramet for each treatment group (**E**). An ANOVA with post-hoc Tukey Test found significant differences between concentrations, allowing us to group the conditions into significance groups (**A**, **B**, and **C**; p = 0.05). A representative image from the scripted analysis of fronds used to generate figures **A** and **E** is shown in panel (**F**). The green circles indicate the ‘fronds’ as interpreted by the analysis algorithm

We used a salt dose response experiment as a basic test of our growth assay workflow. We grew triplicate dishes of *S. polyrhiza* 7498 plants in media supplemented with 0, 50, 100, 150 or 300 mM sodium chloride. We collected images every 2 days over 10 days as well as fresh weight at the start and end of the experiment. In addition, for this experiment we activated an option within the code of the ‘Harvest’ OT (Fig. 4A) to have the technician collect a sample of plants from each plate and manually separate out fronds and take an image (“frond analysis”). Graphical outputs from this experiment are shown in Fig. 4, and were produced using the data analysis scripts available in the Github repository accompanying this paper, along with the image files.

As expected, growth rates decreased proportionally to increasing NaCl concentration (Fig. 4B, C). Additionally, we found no significant difference in frond diameter between test groups (Fig. 4D). During observation, we noticed a change in the density of fronds per ramet across test groups. We were able to easily integrate an analysis into our workflow to interrogate these changes, and found a significant difference in the number of fronds per ramet between 0, 50, and 100–300 mM NaCl (Fig. 4E).

The combination of novel operation types and data analysis scripts facilitated the straightforward collection and analysis of an experiment involving over 165 images of 15 different items as well as additional quantitative data. We propose that these software tools would be useful for the work of other duckweed researchers using similar experimental designs.

**Fig. 5:**
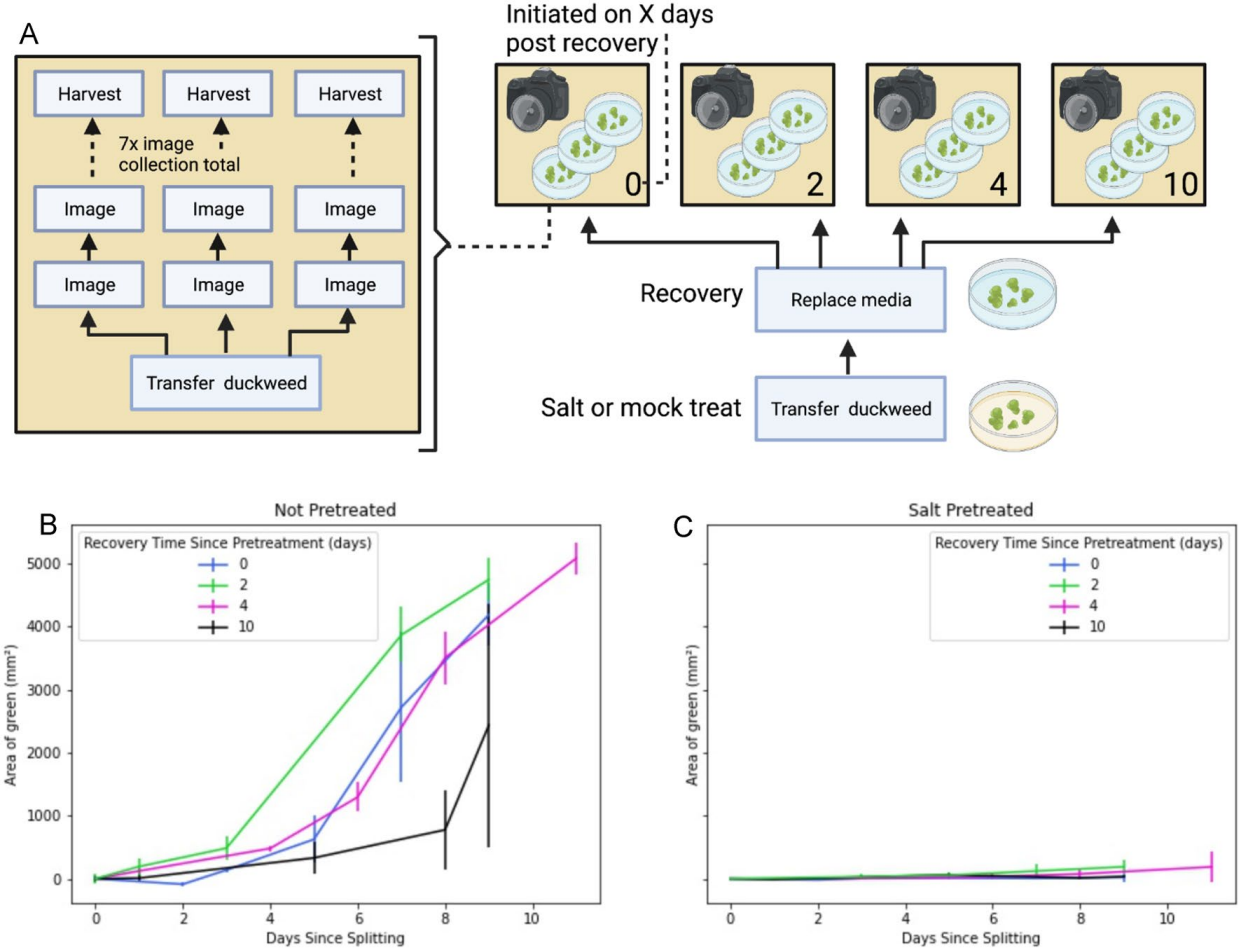
Assessment of duckweed growth rates after variable recovery lengths post salt treatment. An overview of experimental design is shown in (**A**), mirroring the box (operations) and line (items) scheme used in the Aquarium designer interface. Duckweed were grown in dishes containing growth media with 100 mM NaCl or mock for 16 days. At that point the media were replaced, using fresh, salt-free media. At 0, 2, 4 and 10 days after that point, a few ramets were used to initiate a growth assay (yellow boxes) with three replicates and images taken every 1–2 days for 9 days. Due to scheduling difficulties the final image for the ‘4 days’ dishes was taken after 11 days rather than 9 days. Duckweed surface area within each was calculated in each image and results are shown in (**B**) (mock) and **C** (salt-treated) with shared y-axis

**Fig. 6:**
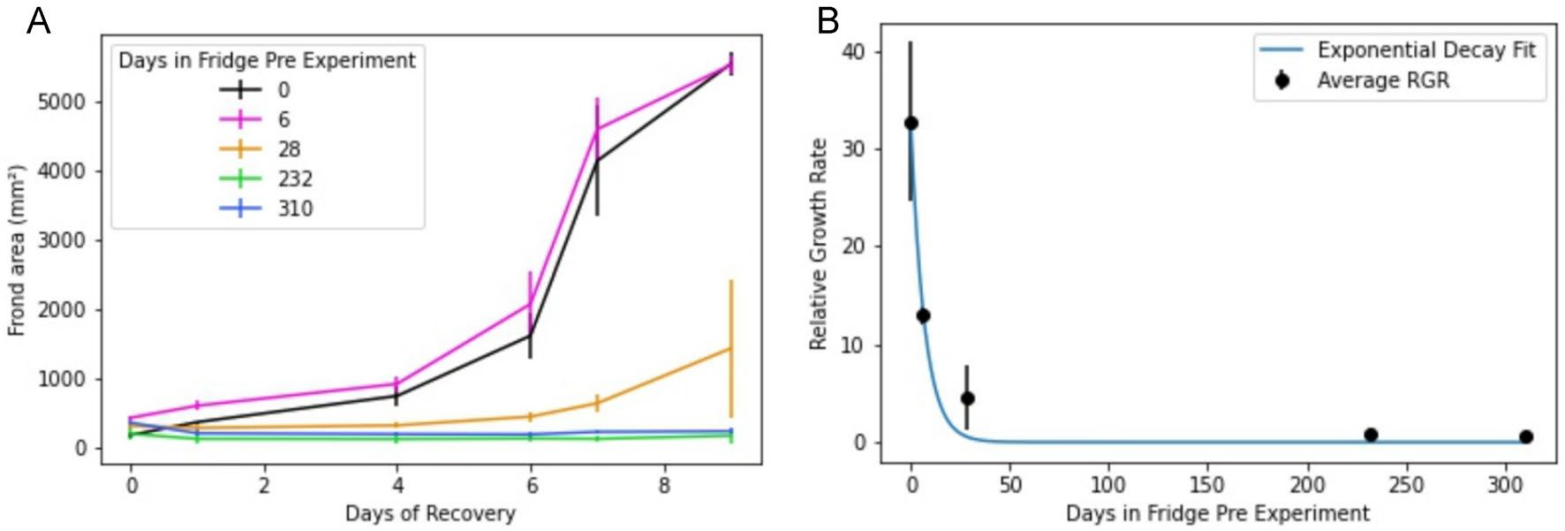
Estimation of maximum duration of fridge storage for healthy recovery. Duckweed plates were stored in a 4 ℃ fridge for varying lengths of time. Fronds were transferred from the stored plates and then allowed to recover in SH4 media over 10 days. Images were taken in approximately 2-day intervals and analyzed to quantify the total area of green duckweed fronds **A**. Values were averaged across biological replicates and plotted. Relative Growth Rates (RGR) for each plate storage time point were calculated by dividing the frond area after 10 days of growth by the initial frond area and error was determined by taking the standard deviation of RGRs across three biological replicates. Calculated RRGs and errors were plotted alongside an exponential decay line of best fit (**B**). The line of best fit in this case suggests that the particular duckweed genotype we studied can be stored under our laboratory conditions at 4 ℃ and remain viable for a maximum of 28 days

We next connected the tools we developed for tracking and managing stock lineages with the growth assay tools described above. We designed and carried out two example experiments: an analysis of the long-term impacts of salt treatment on duckweed populations (Fig. 5), and an experiment to evaluate the impact of fridge storage duration on the growth rates of progeny (Fig. 6).

We began the salt treatment experiment (Fig. 5) by growing two duckweed populations, one in media with 100 mM sodium chloride and a control group grown in salt-free media. Both groups were left to grow for 16 days, and then media for both groups was replaced with fresh, salt-free media. Then, over the course of 2 weeks, a series of growth assays were initiated using a few ramets from the salt-treated or control populations (Fig. 5A). We carried out mock pre-treatments using salt-free media. Duckweed were grown in standard media in petri dishes for 16 days. That mother plate was then used to inoculate new petri dishes filled with standard media in triplicate. That was done at 0, 2, 4 and 10 days after the initial 16 days pre-treatment. That means that the “10 day” mock pretreatment samples (Black line, Fig. 5B) were colonies inoculated from plants that had been in the same media for 16 days, then had their media replaced but remained in the same petri dish for a further 10 days before the start of the experiment, which at that point was fully saturated with fronds. However, even in that case the plants were able to begin dividing and growing within the 10 day growth assay. By contrast, we detected to no increase in total frond area during the 10-day time course for salt treated plants (Fig. 5C), even for the growth assay initiated after 10 days recovery in salt-free media (Fig. 5C).

Next, we set up an experiment to gather data to assess the effects of long-term storage on duckweed viability (Fig. 6). Parent plates were stored in a 4 °C fridge as described in Materials and Methods. This temperature is colder than that typically employed for duckweed cultivation and was chosen due to the logistical constraints of the laboratory equipment available to us. We were therefore curious to measure the impact this cold storage would have on duckweed stock viability. Daughter plates were transferred from stored parent plates that had been stored for 0, 6, 28, 232, and 310 days (Fig. 6A). Growth assays were initiated in triplicate with a few ramets from the parent plate, which was then discarded. We found that plates could be successfully stored for 6 days with no significant impact on growth rate, and that stored plates remained viable for at least 28 days but with a rapid drop off (Fig. 6B). No growth could be detected in growth assays initiated from plates stored for the longest durations of 232 and 310 days (Fig. 6A). Viable storage times are likely to vary based on duckweed genotype and local cultivation conditions. However, using the Aquarium infrastructure, we anticipate similar trials could be run in any lab with relative ease to determine the timeline limitations of specific long-term duckweed storage protocols and ensure consistency between experimental groups initiated from stored duckweed populations.

There are limitations to what can be achieved within the Aquarium system, as explored below. However, we found that being able to both track and forward-define lineages allowed us to reduce the cognitive load and logistical complexity required for multi-day duckweed experiments with large numbers of sample-associated items.

## Discussion

We set out to adapt the existing laboratory management software Aquarium for duckweed protocols with the goal of demonstrating the efficacy and utility of open-source data and metadata management tools for duckweed biologists. We developed new Aquarium OTs to be used in duckweed experimental and maintenance protocols. With these new operations we were able to construct workflows for a variety of experiments; all results in this paper were obtained from combinations of those OTs. We also coded data analysis scripts using Python’s Aquarium API pydent and the open-source image analysis library OpenCV2. We used the scripts to access duckweed images and associated metadata from Aquarium, and then analyze the photographs. Combined, these tools provide a framework for duckweed researchers to address the issues of stock maintenance and experimental data and metadata management. We anticipate continued growth in the applications of image data in plant science and duckweed research, and designed our tools with this in mind. Aquarium, our novel operation types, and the analysis scripts were all created with the intent of being dynamic tools for researchers to tailor and develop for their specific needs in an ongoing process.

In this work, we focused particularly on two possible uses of relevance to duckweed research—managing image data, and tracking lineages. However, we also paid attention throughout the process to other ways that Aquarium supported our work.

The set of types that we have defined in this work can be easily altered or expanded to meet the needs of different users. Explanation of how to create new Aquarium types and other constructs can be found at www.aquarium.bio, along with additional instructions in Additional file 2. For example a particular user may use a different set of item storage systems in their lab and will therefore want to update the Location Wizards. Or they may wish to alter the set of OTs or the Krill code of a given OT. Every research lab is unique, and total standardization is not possible unless all work is carried out by identical robotic automated systems. Accordingly Aquarium was designed to allow for each user to rapidly define types that match their local conditions in order to be able to gain the benefits of standardization, and division of labor, at low financial cost [24]

An unexpected benefit was that Aquarium supported asynchronous cooperative work, as required by the restrictions relating to the COVID-19 pandemic. Aquarium requires work plans to be made explicit and to be composed of modular units that are easily accomplished by an individual technician. This structure was designed with the model of a single lab manager dividing up work among a team of lab technicians all working on site in parallel. It works just as well to support non-hierarchical teams working asynchronously. For instance, while in general each experiment (“Plan” within the Aquarium system) was executed by a single researcher, other personnel may take care of certain tasks within the plan such as collecting image data or transferring duckweed stocks on a particular day. All that needs to be communicated is the Plan ID and the OT name and then all other required information such as item locations and protocol instructions are provided just-in-time by Aquarium and the system logs that the work has been completed as well as when and by whom. Additionally, data collection and data analysis were carried out by separate individuals, using the Trident API (see Fig. 3) to pull relevant data and metadata at the convenience of the analyst without recurring additional communication with the researcher who collected the data.

Extensibility was another feature of Aquarium that proved particularly useful to our work. We were able to rapidly extend our capabilities by generating new OTs or modifying existing ones using the integrated development environment*.* This included the development of the ‘frond analysis’ option within “harvest and record fresh weight” (Fig. 4), which we added after observing differences in frond sizes and fronds per ramet during preliminary experiments. Another example is the ‘record contamination’ operation type that was added during our experiments in response to contamination issues and realizing that we should collect more data to be able to troubleshoot (Fig. 2). We were easily able to extend our Aquarium operation type code base to meet our developing needs during this research project.

Basic research laboratories are dynamic workplaces, with an ever-shifting complement of protocols, tools, materials and project teams. Aquarium was developed within a basic research laboratory setting and was designed to accommodate many of these complexities [24], for instance, its integrated developer environment allows for rapid prototyping and deployment of new protocols, and its modular planning system allows new workflows to be created on the fly. However, during the development and testing of the tools described in this paper we identified challenges that suggest opportunities for the design of software to support work in basic life science research laboratories.

Firstly, we found that Aquarium lacked the flexibility to allow real time responses to conditions on the ground. For instance, halfway through running a job we might find that a particular bottle of media is in fact empty, and there is no way to select a different item from the database without canceling the entire job and starting again. When the real actions deviate from the instructions displayed through Aquarium, more opportunities arise for the actual inventory of the lab to get out of alignment with the digital database. It is well established that plans are not the same thing as real-world “situated actions,” creating difficulties for human–computer interfaces [29]. Providing more opportunities for real-time updating and annotation for Aquarium jobs and plans would allow the benefits of software-supported workflow planning while accommodating the realities of work at the lab bench.

A second limitation of the Aquarium platform is that it represents processes and things well but it doesn’t support the integration of context information and lacks support for direct machine-to-machine communication. For example, it would be useful to be able to connect the environmental condition history (heat, light, humidity) collected by growth chamber sensors with the Aquarium database so that each plant item has an associated set of growth condition data. Similarly, it would be useful to have an easy way to keep track of which machines were used within a particular workflow. A laboratory workflow management system that integrated with sensors throughout the laboratory could, for example, make it easier to troubleshoot when a protocol fails unexpectedly. Similarly, when collecting image data a human technician had to take photos using a camera and then upload the files to Aquarium, creating opportunities for incorrect data associations. A system that acts as a central hub for machines and sensors through the laboratory would better suit the needs of the modern research laboratory.

Aquarium currently supports the definition of tasks and tracks basic data about when a task was carried out and by whom. Task management remains a significant challenge, particular for those wishing to use Aquarium to support cooperative, experimental work. Aquarium was primarily developed around the support of biofabrication workflows rather than experimental workflows, which tend to require planning out a specific series of tasks far in advance. When working with living organisms, the timings between work sessions may have limited flexibility. During work for this study, Aquarium was useful for defining the required tasks ahead of time, but without tools to schedule work ahead of time there was a greater risk of work not getting done at the right time or lack of clarity over who is responsible for what.

## Conclusion

We developed an open-source framework for duckweed image analysis, consisting of the Aquarium code in the supplementary material accompanying this paper (Additional file 1) and the Python scripts publicly available at https://github.com/mtscott321/duckweed_data_analysis. As computational tools and automation become more pervasive in scientific spaces, it is important that we develop the necessary tools to connect software to laboratory management. A crucial aspect of laboratory management software is its dynamism; the software needs to be highly adaptable to accommodate a variety of highly specific experimental designs. As such, this software is most powerful when it is shared open-source, where it can be continually developed and shared between researchers with similar needs. Laboratory management software has the potential to revolutionize the reliability and reproducibility of science done within and between labs, and we strongly encourage every plant scientist to consider integrating LIMS into their research.

## Methods

### Duckweed cultivation

*Spirodela polyrhiza* strain 7498 was sourced from the Rutgers Duckweed Stock Cooperative. Cultures were grown in deep-well (25 mm) petri dishes and containing 30 mL 1.5 g/L × Schenk and Hildebrandt media [30], sourced from PhytoTech Labs, with 0.5% sucrose, adjusted to pH 6. Cefotaxime (10 uM) was added to media after autoclaving to reduce the risk of contamination. When contamination was discovered, plants were sterilized via submersion in 1:10 diluted commercial bleach solution (Clorox) for approximately 60 s, followed by a double rinse with sterile distilled water and then recovery in the aforementioned duckweed growth media. Plants were grown at approximately 28 °C under LED panels with a 12 h photoperiod. Stocks were transferred to fresh mediaevery 2–3 weeks.

Storage plates (Fig. 6) were prepared by transferring roughly 50 healthy ramets onto standard petri dishes containing solid media: 1.5 g/L × Schenk and Hildebrant media without sucrose and supplemented with 1.5 g/L Phytagel (Sigma-Aldrich) and adjusted to pH 6.0. Plates were loosely sealed to allow for gas exchange and placed right side up in a 4 °C fridge. Note that a temperature of 4 °C is not typically used for duckweeds. This temperature was only used due to the equipment available in our laboratory.

### Data collection and analysis

Images of dishes of duckweed were collected at uniform distance using a Google Pixel 3a, then were uploaded as JPEG files to Aquarium. The image collection Aquarium protocol and the accompanying analysis script assume that duckweed are being cultivated in petri dishes or other circular, transparent containers, and that images consist of a single dish on a uniform background.

“Frond dissection” began with random selection of three ramets from a given petri dish. The ramets were placed using tweezers onto a white plastic card, then fronds were manually separated by gently pulling apart using tweezers to rupture the stipe. Any roots were removed and discarded. Isolated fronds were photographed under the same conditions as the petri dishes, then uploaded to Aquarium.

Data was accessed using the pydent API for Python [31] in Jupyter Notebooks. Cultivation images were downloaded to a local machine with accompanying metadata from Aquarium. The images’ resolution was then reduced for easier analysis and the petri dishes were identified using OpenCV2. The images were cropped to the size of the petri dish, and then green pixels were isolated from non green pixels using a manually defined threshold. Green pixels were counted, and then the area was calculated knowing that the diameter of the cropped image was 95 mm—the size of the petri dish. Data were analyzed and plotted primarily using matplotlib, pandas, numpy, and lmfit. All code used in this study is available on Github.

### Aquarium and trident

Aquarium was developed by researchers in the Klavins lab at the University of Washington and has been extensively described elsewhere [24]. Detailed documentation can be found at www.aquarium.bio. Aquarium is open-source, distributed under an MIT license and the code can be found on Github (https://github.com/aquariumbio/aquarium). The work presented in this study was performed using Aquarium release version 2 (latest version as of March 2022—Version 2.7.3). Aquarium is accessed via a web-app with a server accessible to all members of a research group provided with the URL as well as a valid username and password. The Aquarium server used in this study is available to members of the Klavins lab and users of the UW BIOFAB (http://www.uwbiofab.org/) and hosting is provided by Amazon Web Services.

Trident, the Python API for Aquarium, was used in this study to extract data from an Aquarium server. Trident is also available open-source under an MIT license and can be found at PyPI (https://pypi.org/project/pydent/). Trident documentation and installation instructions can be found on Github (https://aquariumbio.github.io/trident/). While dockerized installation can be used to explore the graphical user interface of Aquarium, for use to support research workflows Aquarium must be hosted and accessed via a server. This implementation is, based on our experience, non-trivial and requires a significant time commitment. We recommend that users be familiar with system administration before commencing with local implementation of Aquarium.

### Supplementary Information

**Additional file 1:** Duckweed work operation types (Aquarium file).

**Additional file 2:** S2 - Aquarium Duckweed Workflow - README document.

**Additional file 3:** Overview of Aquarium user experience.

## Data Availability

The datasets generated and/or analyzed during the current study are available in the Github repository, https://github.com/mtscott321/duckweed_data_analysis.
