# Supplementary material for: Open-source workflow design and management software to interrogate duckweed growth conditions and stress responses
Source: Plant Methods. 2023 Sep 1;19:95. doi: 10.1186/s13007-023-01065-3 (PMC10472582; doi:10.1186/s13007-023-01065-3)
Supplement: Supplementary file 2 — Additional file 2: S2 - Aquarium Duckweed Workflow - README document. [file 13007_2023_1065_MOESM2_ESM.docx]

Aquarium Duckweed Workflow - README

The following is intended as a supplement to the existing resources that can be found at [www.aquarium.bio](http://www.aquarium.bio). An overview of the software and why it was created can be found in Vrana *et al*., Aquarium: open-source laboratory software for design, execution and data management, Synthetic Biology, 2021,<https://doi.org/10.1093/synbio/ysab006>.

The instructions below were created in November 2021.

## How to get started with Aquarium

Consider setting up a local instance of Aquarium on your computer using the dockerized installation method outlined on [www.aquarium.bio](http://www.aquarium.bio) ([Direct link to instructions](https://aquariumbio.github.io/aquarium-local/)). This will allow you to fully explore the user interface, create plans, schedule and run jobs as well as import/create/modify operation types and protocol code. In order to proceed from there you will almost certainly want to install Aquarium on a web-accessible server, this could be a virtual server, making use of a cloud computing service, or a local server. In either way if you are working in an institutional setting contact your local IT support for advice and, if required, support with set up.

In parallel to setting up the server you should set up your lab space and management approach ahead of time. The key things to consider are that to make full use of Aquarium’s tools for inventory location management you will want to set aside certain spaces for exclusive use for Aquarium-managed operations. That could mean just a shelf within a fridge, rather than an entire fridge, for example. You can read more about location management at [www.aquarium.bio](http://www.aquarium.bio) or in the paper cited at the top. You will also want to consider whether multiple people will be using Aquarium and if so, how, will you want to separate roles, for instance having dedicated technicians executing lab work. Personnel roles do not need to be hardcoded to an Aquarium instance and you can change your mind as you go, but it’s worth thinking through ahead of time and ensuring that the rest of the team you will be working with an agreed understanding of how Aquarium will be used.

## How to install the duckweed operation types and get started?

The ‘import workflows’ tool found under the burger menu in the top left corner of the GUI can be used to import the set of operation types found in the supplementary files accompanying this manuscript. These “.aq” files include full definitions of *Operation Types,* including the protocol code and also the associated sample types. Note that you are totally free to edit the workflows, and create your own using the built in developer environment (for operation types) or the burger menu to create new sample types or containers.

Further detail on the operation types and libraries included with this manuscript are listed in Table 1 below. Note that, Aquarium requires that all operation types be sorted into ‘Categories’ which are labels used to group operation types. Operation types that are in the same category appear together in the graphical user interface, there is no further functional impact and the category of an operation type can be modified by the user at any time to suit their needs.

Note Ne that the table below does not include the location wizards defined for this study. Location wizards are simple objects within Aquarium, consisting of a vector with a length in 3 dimensions, defining a certain number of spaces that can be filled with items. Items of each container can be assigned to a certain location wizard and if so Aquarium will automatically assign the item to vacant positions in the wizard whenever “.store” is run on that item and will mark the position as vacant whenever “.retrieve” is run on that item. Location wizards can and should be redefined as part of local implementation so that they match the physical realities of storage locations in the user’s laboratory.

| Table 1: Description of Aquarium types included in the .aq file associated with this manuscript | | | |
| --- | --- | --- | --- |
| Type of type | Type Name | Name used in this manuscript (where different) | Additional description / Notes |
| Sample Type | Media |  | Each different media recipe is defined as a different sample of this sample type, and the details of that recipe are stored in the “Plant_Media_Recipes” library, tagged with the appropriate media sample name. |
| Sample Type | Antibiotics |  |  |
| Sample Type | Duckweed line |  | Every genotype of duckweed is a different sample, but every physical manifestation of that genotype is an item within that same sample. |
| Container | 200 mL Agar |  |  |
| Container | 400 mL Agar |  |  |
| Container | 800 mL Agar |  |  |
| Container | Agar Plate Batch |  | A collection, meaning that an item of this type is a way of representing a batch of plates. The number of plates and the media recipe are defined by the user automatically when running “Pour plant media plates” |
| Container | 200 mL Liquid |  |  |
| Container | 4200 mL Liquid |  |  |
| Container | 800 mL Liquid |  |  |
| Container | Batch of plant media or components |  | A more generically named collection. Was used in this manuscript as a way to manage batches of antibiotics to add to duckweed media. |
| Container | Container of Duckweed |  | For the work in this manuscript, ‘container of duckweed’ was physically manifested as a deep well petri dish, but a generic name was chosen to encourage other users to work with the cultivation containers that work best for their purposes. |
| Container | Storage Plate of Duckweed |  |  |
| Operation type | Pour plant media plates |  |  |
| Operation type | Prepare bottles of plant media | Prepare duckweed media (Figure 1) |  |
| Operation type | Add Cefotaxime to autoclaved media | Add antibiotic to media (Figure 2) |  |
| Operation type | Define plate treatment |  | An OT that does not generate any instructions for a technician to execute. It completes automatically as sun as it is run and is a way to add certain conditions to a cultivation experiment, essentially adding tags into relevant database items that can be read by protocol code for downstream operation types and used to modify instructions for technicians as appropriate. |
| Operation type | Harvest and record fresh weight |  |  |
| Operation type | Image container of duckweed |  |  |
| Operation type | Log contamination and discard | Log Contamination (Figure 2) |  |
| Operation type | Record dry weight |  |  |
| Operation type | Replace duckweed growth medium |  |  |
| Operation type | Split duckweed into New containers | Transfer duckweed (Figure 1; Figure 5) |  |
| Operation type | Store duckweed | Store plate of duckweed (Figure 1) |  |
| Library | Plant_Media_Recipes |  | Includes additional protocol code that defines specific media recipes used in duckweed workflows. Referenced by relevant operation types such as “Prepare bottles of plant media” |
| Library | Duckweed Cultivation Library |  | Includes additional protocol code for steps that are used repeatedly in multiple operation types relating to duckweed cultivation. |

## Adapt the protocols to your lab

When making any modifications to any *Types* in an Aquarium server (including operation types, sample types, containers and location wizards) it’s worth considering the consequences, or better yet avoiding any consequences by working in a sandbox Aquarium instance, with a database copied from your live instance ([instructions here](https://aquariumbio.github.io/aquarium-local/)).

- 1. **Create location wizards.** Every *Container* (type of inventory object) has a default location, which could be a simple string such as “Bench” or “Fridge” but can also be a defined Location Wizard, which is a multi-dimensional matrix corresponding to a real physical space e.g. a plant growth chamber might have six shelves, with three trays per shelf and six positions per shelf and would be defined as a 6 x 3 x 6 matrix. Aquarium automatically tries to group inventory together, filling up empty slots in the Location Wizard as they become available. These instructions don’t have to be followed but if they aren’t the reported inventory locations in the Aquarium database will get out of sync with reality. Editing a Location Wizard once in use can have unintended consequences so it’s worth thinking carefully about this before you begin running any Jobs, ensuring that Location Wizards are defined according to the realities of your lab and that default storage for all relevant Containers have been updated.
  2. **Update protocols to match local realities.** Each *Operation Type* in Aquarium is essentially a blueprint for converting defined inputs into defined outputs according to a set of steps defined in the *Protocol* code (written in *Krill)*. Krill is a modified version of Ruby-on-Rails and extensive [documentation can be found here](https://aquariumbio.github.io/aquarium/api/). We deliberately aimed to use generic descriptors for inputs and outputs and to avoid making the protocol instructions very specific to our lab space. However, this wasn’t totally possible and it may furthermore be desirable to you to make the protocols correspond more closely to your lab space and the way you like work to be carried out in your lab. To do this, review the protocol code and make adjustments where desired, making sure to run a test to ensure that the code still executes correctly after making edits. Note that for the operation types in this workflow most of the protocol code is actually housed within accessory libraries which are only protocol code without their own inputs or outputs and can be accessed by multiple operation types. We made extensive use of libraries partly to keep the protocol code pages themselves clean and easy to understand, and also to make it simpler to create new operation types that share certain steps, though currently there are only a few examples of this. You can check the top of each protocol to see which libraries (“needs Category name/library name”) and which specific modules (“import ModuleName”) the *Krill code* used in this protocol can be found in.
  3. **Add images.** Images can be added into a protocol, and will display to the technician when run, which can be useful for providing instructions best delivered visually rather than in text form. To make use of this feature you need to configure a specific folder in the server hosting your Aquarium instance, according to [these instructions.](https://aquariumbio.github.io/aquarium/api/Krill/ShowBlock.html#image-instance_method)

## How to create a plan?

See “Researchers > Designing plans” on [www.aquarium.bio](http://www.aquarium.bio) for a comprehensive introduction to designing plans. Some additional things to bear in mind:

- Many of the operation types in this set rely on *Parameters* to adapt the protocol to the needs of the user e.g. selecting yes for the “cluster analysis” parameter option on ‘Harvest and record fresh weights’ will lead to additional steps within the protocol to take a sample of frond clusters and then manually separate fronds within the cluster and take an image. These options are explained in the “Docs” section of each operation type.
- There are two versions of “Transfer duckweed”. Use “by mass” if you want to start off the duckweed population with a particular mass and/or you want to record initial fresh weight as part of an experiment. Use “by eye” otherwise.
- If you aren’t sure what the exact steps of a protocol are you can use the “TEST” tool in the Developer interface to generate a pseudo job. The instructions won’t render exactly the same as in a real job but this is an easy way to quickly see all the steps of the protocol in human readable format.
- The term ‘workflow’ isn’t found anywhere in the Aquarium software/GUI and you’ll find that every *Plan* in Aquarium is a unique combination of Operation types with specific input and output field values. However, you can copy plans, as well as creating and reusing templates. In addition operation types are grouped into *Categories* and while there is nothing in theory stopping you from mixing and matching operation types from different categories in practice almost all operation types in a given plan tend to be drawn from a single category and so in reality plans tend to be variations on a limited set of recurring patterns, that can be referred to as a workflow.

## How to manage jobs?

A detailed introduction to managing jobs can be found under the “Lab Management” section at [www.aquarium.bio](http://www.aquarium.bio). The most important concept to bear in mind is that a *Job* is a batch of operations belonging to the same operation type that will be run together in one session at the lab bench. These operations do not have to be from the sample Plan or submitted by the same user. The batch could contain only a single operation or a hundred. This is all part of the deliberate abstraction barrier between the design of workflows (Plans) and their execution as part of Jobs. This structure allows for but does not require that someone else other than the workflow planner will actually schedule and execute the jobs. Currently Aquarium doesn’t have in built tools to facilitate communication between those planning workflows and those scheduling and running jobs, that means you need to ensure that any special requests such as specific timing for running a job are communicated to the relevant personnel.

## How to access data and metadata?

Certain metadata are always collected by Aquarium, such as the time and date that each Job was run on, as well as the date that any piece of inventory was created, and all the operations it was used in. However, other data, particularly experimental data need to be uploaded by a user, and ideally this is done following a defined prompt within a Job. Aquarium allows for the upload of text and numbers as well as any arbitrary file during a job. What happens to these inputs once entered or uploaded depends on the corresponding protocol code, which might, for example, ensure that the data are associated with the corresponding output item of the operation and assign a specific key that can be used to retrieve the data later on via a scripted retrieval. While data can be retrieved manually through the GUI, we recommend using the [Trident API](https://aquariumbio.github.io/pydent/), allowing you to use simple Python commands to connect to a specified Aquarium web instance and then retrieve data using a variety of different methods. Generally it is worth thinking ahead of time about the desired data analysis to ensure that not only are the appropriate data and metadata being collected but also to ensure that they will be associated with the inventory/plans/jobs in a way that makes sense for your analysis approach. In the protocol code for the operation types shared in this manuscript the keys used for data associations are listed at the top of each protocol, so as to avoid the situation that you have to carefully comb through the protocol code of a given operation type to find out where your data of interest got associated and how to retrieve it.
