# Supplementary material for: Open-source workflow design and management software to interrogate duckweed growth conditions and stress responses
Source: Plant Methods. 2023 Sep 1;19:95. doi: 10.1186/s13007-023-01065-3 (PMC10472582; doi:10.1186/s13007-023-01065-3)

Within the Aquarium GUI new Operation Types are created and defined through the 'Developer' tab.

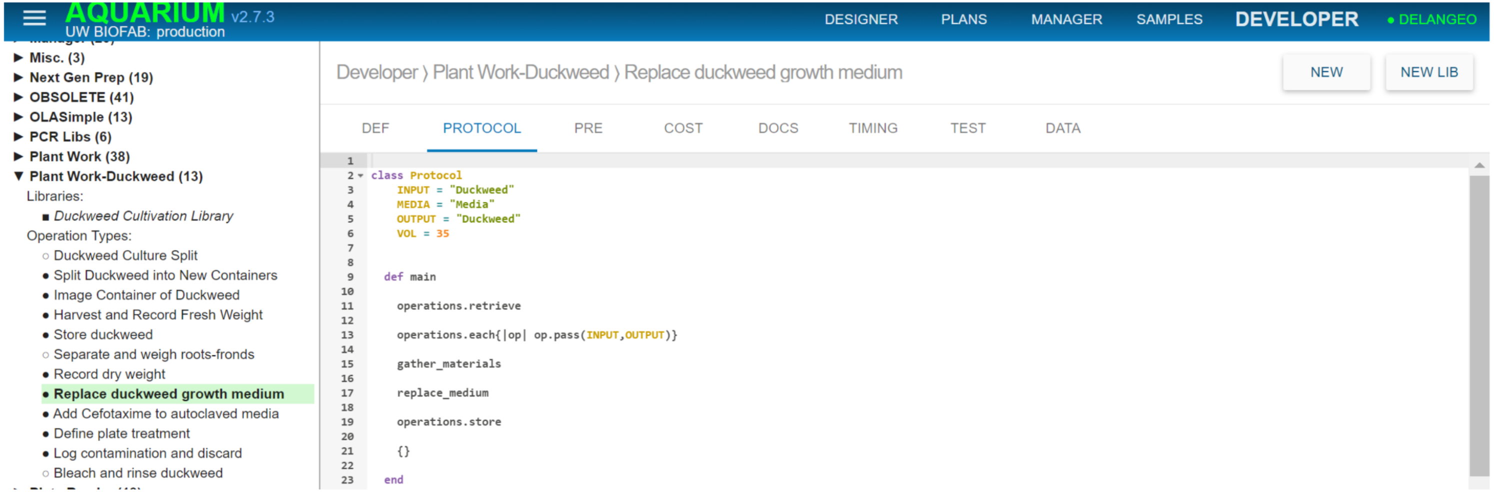

The instructions and calculations of the protocol are defined using *Krill* (a derivative of Ruby on Rails, either directly through the GUI or by importing code developed elsewhere

```
class Protocol
    INPUT = "Duckweed"
    MEDIA = "Media"
    OUTPUT = "Duckweed"
    VOL = 35


  def main

    operations.retrieve

    operations.each{|op| op.pass(INPUT,OUTPUT)}

    gather_materials

    replace_medium

    operations.store

    {}

  end

  def gather_materials

      media_bottles = operations.map{|op| op.input(MEDIA).item}.uniq
      pipettes = "#{media_bottles.length + 1} 50 mL Seriological pipettes"

      show do
          title "Gather materials"
          note "Gather following materials and transfer to balance area"
          note "Bottles of media"
          media_bottles.each do |b|
              check " #{b.sample.name} #{b.id} from #{b.location}"
          end
          note "Plates of duckweed"
          operations.each do |op|
              op.output(OUTPUT).item.id
          end
          check pipettes
          check "Empty container of same type used to grow duckweed, to be a blank"
      end
  end
```

Yellow uppercase used to define constants that can be used later in the protocol

Between "def main" and "end" the elements of the protocol are defined, with details for each element are provided below

Calculations can be integrated into protocols. In this case, connecting the number of required seriological pipettes to the number of media_bottles being handled plus one.

"operations" is a Krill method that acceses a list of details for all the specific operations in the Job. "Each.do" is standard Ruby on rails to iterate through a list.

In the "Designer" tab any user can create custom workflows by combining available operation types. These workflows are called *plans*. Each instation of an operation type within a plan is an *operation*

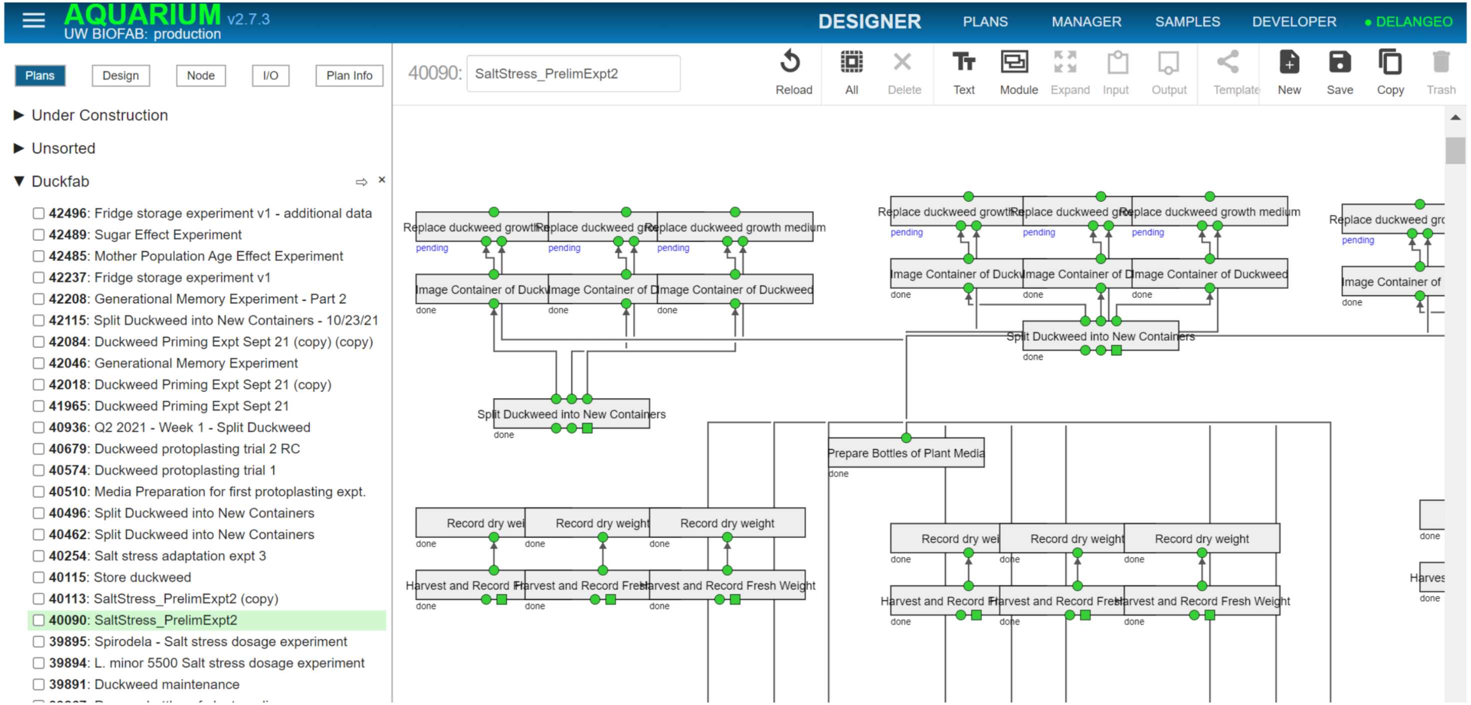

Before a plan can be launched inventory must be selected for all inputs that aren't created during the lifetime of the workflow. Inventory information, including item IDs, can be looked up through the Samples page as well as pop up windows in other pages.

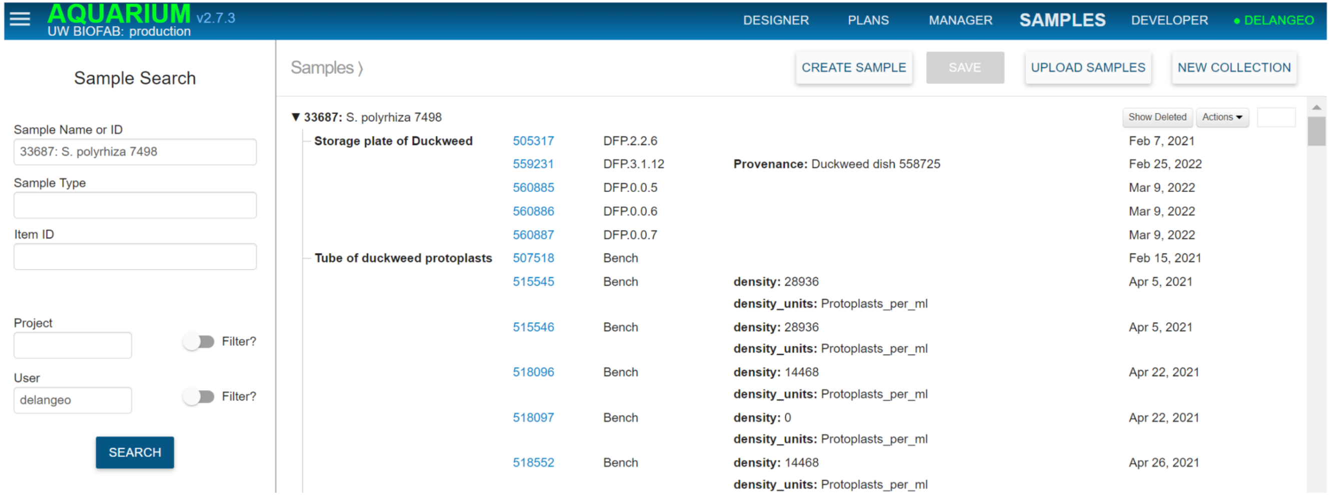

Once a plan is launched all its constituent operations become available in the Manager page. Operations of the same type can be batched together to create a *Job.*

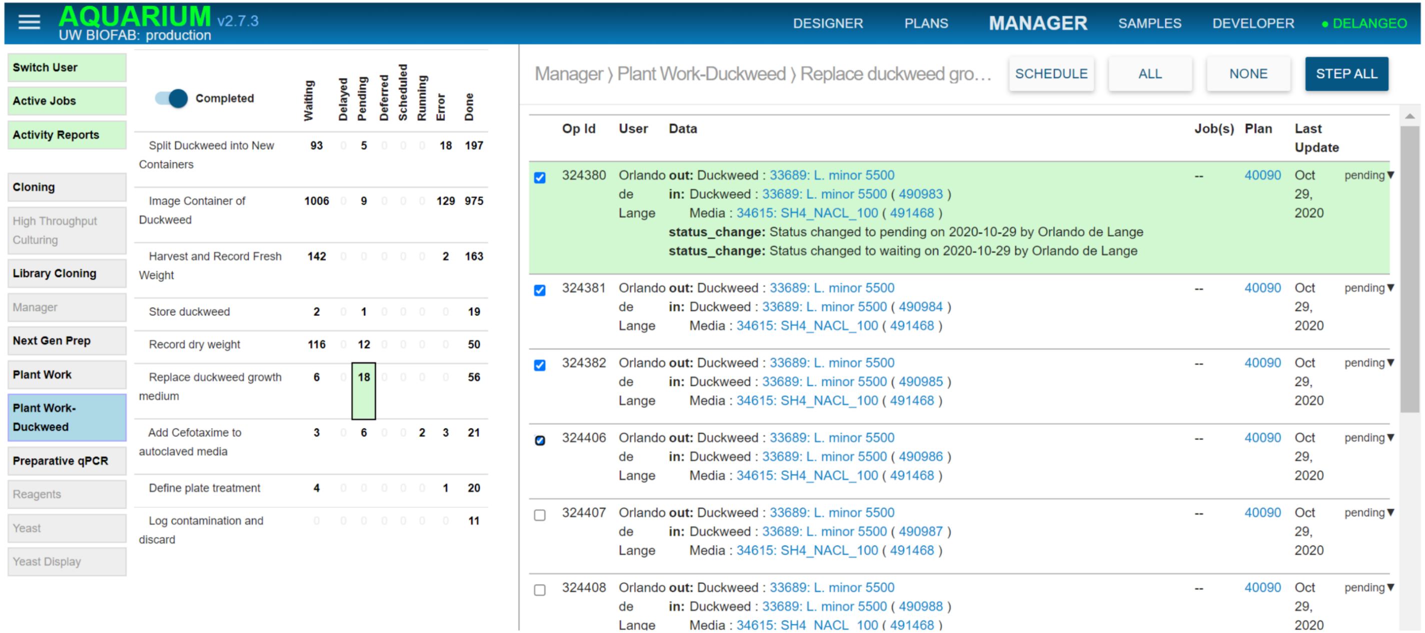

When a job is run the protocol code is parsed to create a set of interactive on-screen instructions to be followed and responded-to by whoever is in the lab to run that job on the day

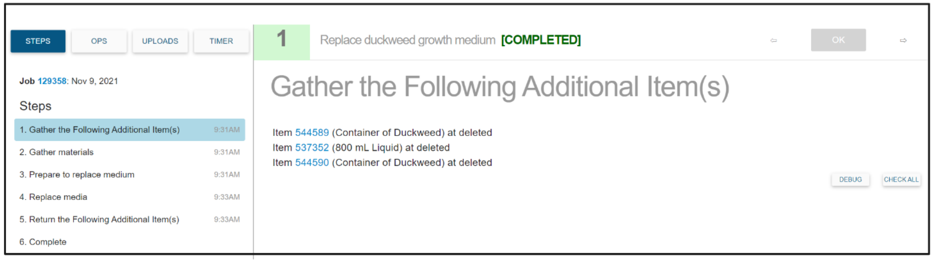

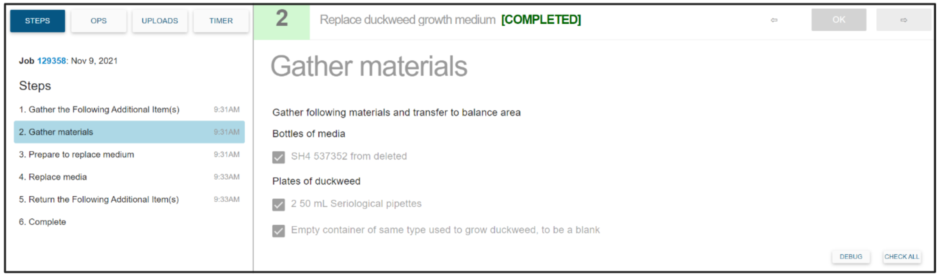

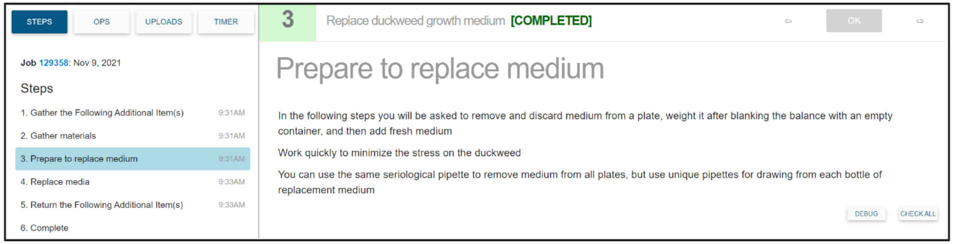

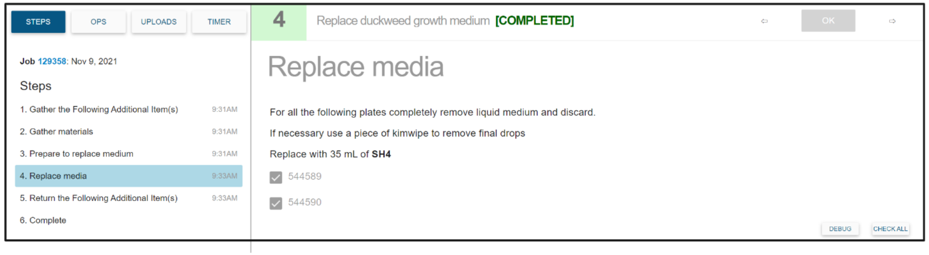

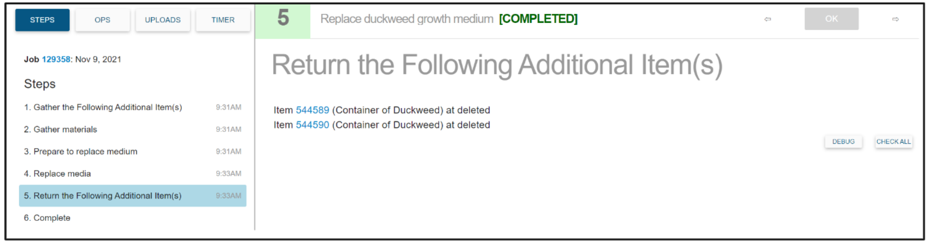

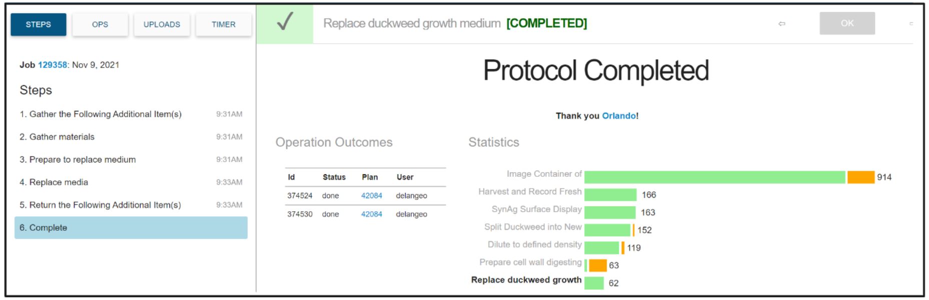

Supplement: Supplementary file 3 — Additional file 3: Overview of Aquarium user experience. [file 13007_2023_1065_MOESM3_ESM.pdf]
